# Intraoperative Polymerase Chain Reaction from Cardiac Valve Tissue Is Beneficial for Guiding Further Therapy in Patients with Infective Endocarditis

**DOI:** 10.3390/jcm13154319

**Published:** 2024-07-24

**Authors:** Mascha von Zeppelin, Seyed Arian Gharoony, Zdenka Holubcova, Razan Salem, Jan Hlavicka, Stephan Heyl, Marco Ochs, Thomas A. Wichelhaus, Johanna Kessel, Anton Moritz, Thomas Walther, Tomas Holubec

**Affiliations:** 1 Department of Cardiovascular Surgery, University Hospital Frankfurt, Goethe University Frankfurt, 60596 Frankfurt/Main, Germany; mascha.vonzeppelin@unimedizin-ffm.de (M.v.Z.); arian.gharoony@gmx.de (S.A.G.); zdenka.holubcova@unimedizin-ffm.de (Z.H.); razan.salem@unimedizin-ffm.de (R.S.); jan.hlavicka@unimedizin-ffm.de (J.H.); moritzanton@web.de (A.M.); thomas.walther@herz-frankfurt.de (T.W.); 2 Department of Internal Medicine III, Cardiology, University Hospital Frankfurt, Goethe University Frankfurt, 60596 Frankfurt/Main, Germany; stephan.heyl@unimedizin-ffm.de (S.H.); marco.ochs@unimedizin-ffm.de (M.O.); 3 Institute of Medical Microbiology and Infection Control, University Hospital Frankfurt, Goethe University Frankfurt, 60596 Frankfurt/Main, Germany; thomas.wichelhaus@ukffm.de; 4 Infectious Diseases Unit, Department of Internal Medicine II, University Hospital Frankfurt, Goethe University Frankfurt, 60596 Frankfurt/Main, Germany; johanna.kessel@ukffm.de

**Keywords:** infective endocarditis, surgical therapy, blood culture, sample culture, polymerase chain reaction (PCR)

## Abstract

**Background**: Infectious endocarditis (IE) remains a critical condition despite all the medical advances in recent decades. Reliable pathogen identification is indispensable for precise therapy. The aim of this study was to evaluate the diagnostic and therapeutic benefit of additional polymerase chain reaction (PCR) in comparison with microbiological culture alone based on intraoperative tissue sampling for patients operated on due to IE. **Methods**: A total of 224 patients diagnosed with acute or subacute IE were analyzed. Intraoperatively resected infectious tissue was analyzed using both PCR and microbiological culture. Subsequently, the results of the detection of bacteria obtained based on intraoperative measurements from tissue via culture and PCR were compared with preoperative blood culture results. Furthermore, we evaluated the therapeutic impact of the culture and/or PCR results obtained from cardiac tissue. **Results**: The 224 patients were 63 ± 17 years old, and 64 (29%) were female. In total, 149 (67%) suffered from aortic valve endocarditis, 45 (45%) had mitral valve endocarditis, and 39 (18%) were afflicted with double-valve endocarditis. Prosthetic valve endocarditis was present in 70 (31%) patients. Pathogens were detected in 70% of the cases analyzed via PCR using cardiac valve tissue and in 25% of those analyzed via a culture of cardiac valve tissue; this figure was only 64% for preoperative blood culture. Overall, a pathogen was identified in 197 patients (88%), leading to antibiotic therapy. Targeted antibiotic therapy, based on the PCR results, was carried out in 37 cases and was conducted based on a culture from cardiac valve tissue in three cases. Finally, in 12% of patients, the causative pathogen remained unclear. **Conclusions**: For patients suffering endocarditis, PCR analysis is indispensable and superior to preoperative blood culture and intraoperative culture in detecting bacteria. Based on PCR testing, antibiotic therapy can be individually adjusted. The high precision of pathogen identification may lead to a significant reduction in IE-associated morbidity and mortality.

## 1. Introduction

The incidence of infective endocarditis (IE) has remained roughly stable over the past 30 years; however, clinical management is a challenge even in industrial countries due to increasing risk factors such as an aging population, diseased or replaced heart valves, intravenous drug abuse, diabetes mellitus, hemodialysis, and intracardiac devices [1,2,3]. Despite the great advances in medicine in recent decades, the morbidity and mortality of IE remain significant [4,5]. In addition, more than 40% of patients with IE require cardiac surgery, which is associated with substantial surgical morbidity and mortality (up to 30%) [2,4]. Pathogen detection and, if possible, antibiotic susceptibility testing are imperative for optimal antimicrobial therapy for IE [6]. This is currently and predominantly performed using preoperative blood and intraoperative tissue sample cultures. However, several factors can lead to sterile cultures (such as prior antibiotic therapy, incorrect sampling, or intracellular slowly proliferating bacteria), requiring alternative diagnostic tools to identify the causative microorganism [2,3,7,8]. Broad-range 16S rDNA PCR based on surgically removed valve tissue followed by DNA sequencing may provide additional diagnostic sensitivity [9], especially for patients with blood-culture-negative endocarditis [10,11,12,13,14,15].

For patients with blood-culture-negative endocarditis, additive blood tests such as a specific PCR analysis and a serological test should be performed [16]. In some cases, the superiority of the PCR testing of blood over 16S rDNA analysis has been described. For example, Fournier et al. described the detection of *Coxiella burnetii* at a sensitivity level of 53% in a PCR analysis of blood, whereas a sensitivity of only 14% was achieved with 16S rDNA [11]. If *Tropheryma whipplei* is considered, pathogen detection can be increased to 100% by PCR analysis of the valve [17]. However, PCR testing also has its limitations: it is not suitable for testing for the entire spectrum of pathogens. Moreover, 16S rDNA testing in blood samples has limited sensitivity, which has led many healthcare facilities to forego the routine PCR testing of blood for infectious endocarditis cases. 

To date, intraoperative culture has been the gold standard, but it is limited by the above-mentioned limitations [11]. Therefore, intraoperative PCR analysis can provide valuable information regarding IE. PCR analysis is a rapid diagnostic method that is independent of growth [12].

While the American Heart Association recognizes PCR analysis of the affected heart valve without further specification [8], the European Society of Cardiology recommends specific PCR testing of both blood and valve tissue [16]. The British Society for Antimicrobial Chemotherapy considers PCR testing of the valve tissue to be a minor Duke criterion [18]. 

The aim of this study is to assess the diagnostic benefit of PCR in comparison to cultures from valve tissue for patients undergoing cardiac surgery for IE and its potential influence on postoperative antimicrobial therapy.

## 2. Materials and Methods

### 2.1. Study Design

From January 2010 to December 2017, a total of 389 patients who underwent valve surgery due to (suspected) acute or subacute IE were identified in our prospective database. The diagnosis of IE was based on Duke criteria [19]. Of these 389 patients, we included all 224 patients who presented with preoperative microbiological blood cultures and who underwent 16S rDNA PCR testing of their removed valve tissue. Exclusion criteria were chronic endocarditis and conservative treatment, as well as missing intraoperative tissue samples.

This study was approved by the Institutional Ethical Committee (reference number: 61/18; date of approval: 14 March 2018), and the need for informed consent was waived due to the retrospective nature of this study.

### 2.2. Surgical Procedure

Surgical access was either via conventional median sternotomy or partial upper sternotomy (J-cut into the 4th intercostal space). In one patient, an antero-lateral mini-thoracotomy was performed. Cardio-pulmonary bypass (CPB) with standard cardioplegic cardiac arrest was applied in all patients.

Arterial cannulation for CPB was performed either via the aorta, femoral artery, or subclavian artery. Venous cannulation was carried out either via the right atrium, bicaval, or femoral vein. After the establishment of CPB, the aorta was clamped and blood cardioplegia administered. Depending on the extent of the endocarditis, resection and repair of the diseased tissue were carried out; additional procedures, e.g., replacement of the aortic root, were performed when indicated. Abscesses were drained surgically, and cardiac structures were repaired using patches whenever required. After successful surgical correction, cardiac reperfusion was initiated, starting with a hot shot for 3 min and then the release of the aortic cross-clamp. After transesophageal echocardiographic control, the patient was weaned from CPB, and the chest was finally closed in the standard fashion. Transesophageal echocardiography was routinely used to assess valve competence.

### 2.3. Microbiological Methods

After intraoperative collection, valve tissue samples were split into two samples, one for culture and the other for PCR. Tissue samples were homogenized prior to culture preparation. Culture was carried out on solid culture media (i.e., blood agar aerobic, blood agar anaerobic, chocolate blood agar 5% CO_2_, MacConkey agar, and Sabouraud agar; Oxoid, Wesel, Germany) using a three-phase plating technique. Tissue homogenate was additionally inoculated into liquid cultures (i.e., brain heart infusion and thioglycolate broth; Oxoid, Wesel, Germany). Incubation was carried out at 36 ± 1 °C for 14 days [20].

For PCR, tissue samples were digested with tissue lysis buffer and proteinase K and then subjected to DNA extraction using a commercially available kit (Roche, Mannheim, Germany). The identification of bacteria by PCR involved amplification and sequencing of the 16S rRNA gene with primers (Molzym GmbH&Co, Mastermix 16S complete bak, Bremen, Germany) targeting the 3′-end of the 16S rDNA (16 S-F, 5-CAAACAGGATTAGAGATACCC) or the 5′-end of the 16S rDNA (16 S-R, 5-CCCGGGAACGTATTCACCG), as described previously [17] (National Library of Medicine, BLAST, Rockville Pike, Bethesda, MD, USA). For real-time PCR (LightCycler, Roche Deutschland Holding GmbH, Germany), the following parameters were used: initial denaturation at 95 °C for 2 minutes, followed by 40 cycles of denaturation at 95 °C for 5 seconds, annealing at 55 °C for 20 seconds, and extension at 72 °C for 30 seconds [20] (Biorad CFX Opus Real-time PCR System, Bio-Rad Laboratories. Inc, Hertfordshire, England and Wales).

### 2.4. Statistical Analysis

The data were entered into a database using Microsoft Office Excel^®^ (version 2010 for Windows; Microsoft Corp, Redmond, WA, USA). The Kolmogorov–Smirnov test was used to assess the normal distribution of data. Continuous variables are reported as the mean ± standard deviation or the median and range for non-normally distributed data. Categorical variables are reported using the number and percentage of observations. Pearson’s chi-squared test was used to compare categorical variables. Statistical analysis was performed using Microsoft Office Excel® (version 2010 for Windows; Microsoft Corp, Redmond, WA, USA) and BIAS software (version 11.08 for Windows; Epsilon Verlag GbR Hochheim Darmstadt, Germany, 1989–2020), in which a *p*-value below 0.05 was considered statistically significant.

## 3. Results

### 3.1. Study Population and Clinical Data

From January 2010 to December 2017, 389 patients suspected of exhibiting acute or subacute IE underwent cardiac surgery. Of these, 224 patients fulfilling the inclusion criteria were included in this study, with a mean age of 63 (range 23–84) years; 71% (*n* = 160) were male and 29% (*n* = 64) were female (Table 1).

From 143 patients, a pathogen was detected in blood cultures, where 36% (*n* = 81) of the patients remained without bacterial detection until surgical treatment. In 29% (*n* = 66) of patients, the focus of infection could be identified: 24% (*n* = 16), oral/dental; 18% (*n* = 12), spondylodiscitis; 18% (*n* = 12), intravenous drug abuse; and 6% (*n* = 4), abscess of the soft tissue or lead infections in pacemaker therapy. Other suspected causes included catheter infections and urinary tract infections, including urosepsis, gastroenteritis, or pneumonia; however, these were very rare.

In the preoperative transesophageal echocardiography, vegetation of the aortic valve was detected in 40% (*n* = 89) of cases, abscesses in 8% (*n* = 19), and a combination of both in 13% (*n* = 29). With regard to the mitral valve, vegetation occurred in 40% (*n* = 90), abscesses in 1% (*n* = 2), and a combination in 3% (*n* = 7). Regarding the tricuspid valve, vegetation was detected in 8% (*n* = 18) (Table 1). The median EuroSCORE II was 8.35 (11.99).

A proportion of 67% of patients had an indication for operative aortic valve treatment, among which 37% had underlying valve disease: 63% had aortic valve insufficiency, 7% had aortic valve stenosis, 9% had a combined valve pathology, and 18% had preoperative endocarditis-related embolization. Sepsis was present in 4% of cases.

**Figure 1:**
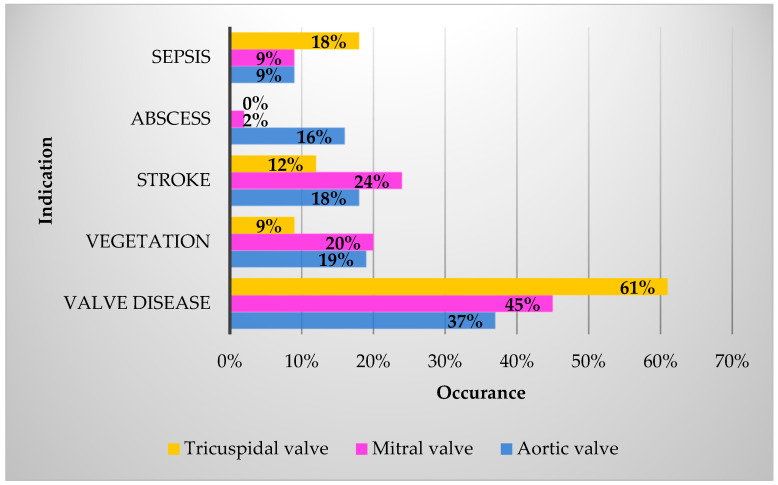
Indication for valve surgery; valve disease: insufficiency, stenosis, or combined.

A proportion of 45% were indicated for mitral valve surgery, with 45% due to valve disease: 82% due to insufficiency, 2% due to stenosis, and 1% due to a combined pathology. Embolization occurred in 24%, sepsis occurred in 9%, and 15% had tricuspid valve endocarditis. A proportion of 69% of IE affected the native valves, and 31% represented prosthetic valve endocarditis (Figure 1). A proportion of 18% had double valve endocarditis, and abscesses were present in 27%.

Aortic valve repair was performed in 22% (*n* = 37) of patients and valve replacement was necessary in 78%: biological prosthesis, 39% (*n* = 66); biological valved-conduit, 25% (*n* = 42); mechanical prosthesis, 11% (*n* = 18); and mechanical valved-conduit, 5% (*n* = 8). In mitral valve IE, a repair was performed in 68% (*n* = 74) of patients, while 32% (*n* = 35) underwent a valve replacement. In the case of a tricuspid valve, a valve-sparing repair was carried out almost exclusively.

During the study period, our institutional protocol did not dictate the submission of intraoperative samples for histopathological examination, as is the case today. Therefore, only 49.95% of the cases (111 patients) underwent histopathological assessment of their removed valve tissue. Of these, acute or subacute endocarditis was detected in 87 patients (78.3%), and a chronic inflammatory process was detected in 9 (8.1%). In 15 (13.5%), there was no histopathological evidence of an inflammatory process.

Abscessing was present in 27% (*n* = 60) of the cases. A complex reconstruction had to be performed in 20% (*n* = 45) and a simple abscess was covered in 6% (*n* = 14).

**Figure 2:**
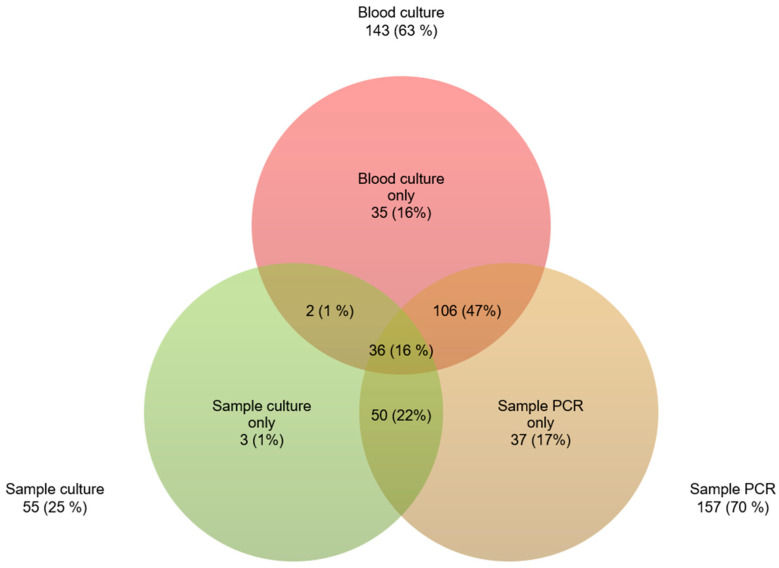
Venn diagram comparing detection by each microbiological method; all positive results.

Of the samples taken intraoperatively, 157 (70%) were PCR-positive and 55 (25%) were culture-positive. The common intersection for PCR- and culture-positive samples was 50 detections. This means that 48% (*n* = 107) of the pathogens were detected by PCR and not by culture. In contrast, there were only 2% (*n* = 5) more detections in the culture of intraoperative tissue than in the PCR. With the help of these two methods (intraoperative sample: PCR and culture), a pathogen determination could be carried out in 162 (72%) of intraoperatively obtained tissue samples. Including the preoperative blood cultures, only 12% (*n* = 27) of endocarditis remained without pathogen detection. With regard to the blood cultures determined to be negative preoperatively (36%; *n* = 81), microorganisms could be detected in 67% (*n* = 54) by means of PCR from intraoperative tissue (Figure 2).

Regarding the intraoperatively obtained cultures and the PCR analysis, a statistically significant correlation was found (*p* < 0.001). No statistical significance was found between PCR analysis and preoperative blood culture diagnostics (*p* = 0.089), as well as in the comparison between intraoperative cultures and blood culture analysis (*p* = 0.48).

*Staphylococcus aureus* (32%), viridans streptococci (17%), and *Enterococcus faecalis* (14%) were the most frequently detected pathogens (Table 2). Pathogens were detected in 10% of cases, such as *Staphylococcus epidermidis*, *Propionibacterium acnes*, *Streptococcus agalactiae*, and *Granulicatella adiacens* (Table 2).

Pathogens were detected both in the preoperative blood culture and in the intraoperative PCR in 106 patients. Of these, 96 were identical, but different pathogens were detected in 14. This resulted in a potential contamination rate of 13%. However, it remains unclear whether the contamination originated from the PCR sample or from the blood culture, or even whether a multiple bacterial spectrum was involved.

In 81 patients (36%), no pathogens could be detected preoperatively by the blood culture. Based on the intraoperatively obtained results, especially PCR testing, the antibiotic therapy was changed in 54 patients (24%). The PCR result was positive in 51 patients (23%), while the preoperative blood culture was negative. In 17 patients (8%), pathogens were detected in the intraoperative culture, while the blood culture was negative. After deduction of the common intersection, 17% (37) of the therapy changes were attributable to the PCR and 1% (3) to the intraoperative culture (Figure 2).

Some pathogens are difficult or impossible to grow in culture, e.g., *Bartonella* species. Endocarditis patients with these pathogens benefit from PCR, as reliable detection can be achieved. In our study, this applied to two patients, in whom *Bartonella quintana* and *Bartonella henselae* could be detected by PCR analysis.

### 3.2. Early Clinical Data

Within the first 30 days, 15% (*n* = 33) of the patients died. Seventeen patients underwent surgical treatment due to an abscess, which resulted in an extensive procedure. Causes of death were multi-organ failure in 58% (*n* = 19), low-cardiac-output syndrome in 18% (*n* = 6), sepsis in 9% (*n* = 3), cerebral embolism in 6% (*n* = 2), and low-cardiac-output syndrome following intraoperative biventricular failure in 1 case.

A total of 43 (19%) patients needed postoperative re-exploration due to bleeding, 2 (6%) suffered from a stroke, and 65 (29%) required renal replacement therapy postoperatively. A total of 25 (11%) patients required permanent pacemaker therapy for higher-grade AV-block, and a postoperative pericardial effusion requiring treatment occurred in 36 (16%).

## 4. Discussion

The growing prevalence of endocarditis can be attributed to the aging population, increased life expectancy, increased prevalence of degenerative cardiovascular diseases, and a higher rate of implanted medical devices [21]. Concurrently, the number of pathogens (multiresistant *Staphylococci* and *Enterococci*, mycoses, etc.) has been growing, making therapy increasingly difficult [6]. The pathogen spectrum has also adapted to medical progress, with *Staphylococcus aureus* in particular on the rise in industrialized countries. An association of staphylococcal infections consisting of chronic hemodialysis, diabetes mellitus, and intravascular access has been described [6]. Our data also show an increase in IE with *Staphylococcus aureus* during recent years, which is also due to the increase in the nosocomial infection rate [6].

Infective endocarditis is associated with severe and often fatal complications. In the literature, cerebral impairment is listed as the most devastating and, at the same time, the most common of all extracardiac complications, with an incidence of 25–56% [22]. In our cohort, 79 IE patients were referred to surgery after the onset of neurological symptoms. Two patients suffered from periprocedural stroke with a poor prognosis. Furthermore, there is a considerable risk of embolism in large (>10 mm), mobile vegetation of the left-sided valves, which is often associated with *Staphylococcus aureus*. This can be significantly reduced by early and pathogen-specific antibiotic therapy [23].

As early symptoms of IE may be vague and non-specific, early diagnosis and appropriate therapy remain a challenge. This can be improved by means of sufficient bacterial detection. In this study, about one-third of the blood cultures examined were negative, which is slightly higher than the value described in the literature [24]. In the majority of patients in this study, empiric antibiotic therapy was initiated before blood cultures were collected, which may have had a negative impact on the blood culture results. For this reason, pathogen specification is essential, and detection by PCR can be life-saving in this regard.

The great advantage of the PCR method with detection of the 16S rRNA gene is the detection of pathogens under antibiotic therapy, as well as in the case of detection of a spectrum that is slow and difficult to grow. Various studies have already demonstrated the superiority of the PCR method over conventional cultures for highly pathogenic microorganisms, such as *Coxiella burnetti* or *Tropheryma whipplei* [25,26]. Nevertheless, there is no substitute for the adequate collection of blood cultures before the start of antibiotic therapy, which remains the gold standard. A disadvantage of PCR is that it does not provide information on the sensitivity to antibiotics, which limits the information that can be used to guide treatment. Cultures obtained intraoperatively have limited validity, as antibiotic treatment has often been initiated prior to intraoperative sample collection. Furthermore, a positive blood culture or a positive valve tissue sample at the time of surgery has an influence on the duration of therapy, as it translates to an active IE.

For native valve tissue samples, the sensitivity, specificity, and positive and negative predictive values of 16S rDNA PCR are reported as 94.1, 100, 100, and 90%, respectively. The same values for traditional cultivation are reported as 17.6, 88.9, 75, and 36.4%, respectively [27]. One limitation is the risk of contamination of the samples and PCR inhibitors, which may be present in the tissue samples or in the transport medium [28,29,30].

In accordance with the current guidelines, a calculated, empirical antibiotic therapy is initiated in IE before the blood culture results arrive [2]. As soon as the results are available postoperatively or by positive blood culture, it is adapted to the pathogen after consulting the infectious disease specialist. In this study, there were 81 patients in whom no bacterial detection could be carried out preoperatively. In 54 of these patients, intraoperative testing was able to detect the pathogen, which resulted in a change in antibiotic therapy. In detail, 51 (23%) of the positive results were based on PCR testing and 17 (8%) on cultivation. Subtracting the common intersection (*n* = 14), PCR testing resulted in a change in antibiotic therapy in 37 cases and culture in 3. In this study, we were also able to demonstrate a statistically significant superiority of the intraoperative PCR over the intraoperatively obtained cultures (*p* < 0.001). In contrast, our study showed no statistically significant superiority of PCR over preoperative blood culture analysis (*p* = 0.089). This underlines the gold standard of blood culture collection. 

In the present study, the additional value of PCR analysis over cultivation from intraoperative tissue samples was clearly demonstrated: 70% of the analyzed samples could detect pathogens by molecular genetics testing. This is in contrast to culture cultivation, which was only able to detect 25% of the pathogens. This corresponds to an increase of 107 pathogen determinations on the PCR side compared to the culture detection and underlines the added diagnostic value.

Various studies have shown that molecular biological detection, via PCR, from intraoperative material significantly improves diagnostic performance. The sensitivity has been documented between 41 and 96%, and the specificity between 90 and 100% [31]. The current IE guidelines already include the 16S PCR analysis. This applies in particular to patients who have negative preoperative blood cultures. Most laboratories today have the technology to carry out PCR testing. However, this recommendation does not currently apply to all endocarditis patients.

### Study Limitations and Strengths

This study provides data from a long time period (over seven years) and a large number of patients surgically treated due to IE. The results of our study may contribute to a consensus on the effectiveness of PCR samples in endocarditis patients and promote the broader adoption of this effective diagnostic tool in these patients.

This study has also several limitations. This is a retrospective, single-center study and all inherent drawbacks apply. The follow-up was only short-term. Although the inclusion criteria were applied, the subjective decision making of the surgeons involved could not be excluded, based on their personal experience. In addition, the study period was several years ago, which might not have included the most recent medical and surgical practices. For example, it is now common practice to perform a histopathological examination of tissue obtained intraoperatively. Our study included an all-comer population including high-risk patients, which might have influenced the postoperative outcome.

Future studies should be multicenter in order to include a larger number of patients. A prospective, possibly randomized, study would be beneficial over a retrospective evaluation. Furthermore, the diagnostic tools should be defined in advance in order to generate as few variables as possible. In addition, a longer-term follow-up would be preferable (e.g., 10-year mortality). More advantages for diagnosis using other detection methods could then possibly be provided.

The results derived can help physicians especially in the treatment of infectious endocarditis.

## 5. Conclusions

16S rDNA PCR is indispensable in cardio-surgical patients with suspected IE who have been previously treated with antibiotics without any bacterial detection. Due to the PCR results, surgically treated IE patients can further be treated with antibiotics in a targeted manner postoperatively. PCR is also a valuable tool regarding a reduced development of antibiotic resistance. PCR analysis may contribute significantly to the reduction in high mortality and should be established as a gold standard diagnostic tool in surgically treated patients due to IE. Nevertheless, PCR diagnostics should not replace the current gold standard: the preservation of at least three blood cultures before starting antibiotic therapy. Further investigation is warranted, ideally by means of randomized control studies.

## Figures and Tables

**Table 1:** Preoperative demographics and clinical data.

Demographics	*n* (%)
Number of patients	224
Age, median (range), years	63 (23–84)
Gender	
Male	160 (71%)
Female	64 (29%)
Comorbidities	
Stroke	79 (35%)
Coronary artery disease	56 (25%)
Diabetes	21 (9.45%)
NYHA class	
III	104 (46%)
IV	70 (3%)
LV ejection fraction	
>50%	155 (69%)
30–50%	67 (20%)
<30%	2 (1%)
Kidney injury (KDIGO)	
Stage G1 (GFR ≥90 [mL/min/1.73 m^2^])	67 (30%)
Stage G2 (GFR 60–90)	70 (31%)
Stage G3 (GFR 30–60)	63 (28%)
Stage G4 (GFR 15–30)	16 (7%)
Stage G5 (GFR <15)	8 (4%)
Preoperative dialysis	11 (5%)
EuroSCORE II	8.35 (11.99)
Endocarditis	
Native	154 (67%)
Prosthetic	70 (31%)
Aortic valve	
Insufficiency (grade I–IV)	140 (63%)
Insufficiency ≥ grade II	102 (46%)
Stenosis	15 (7%)
Combined	21 (9%)
Not affected	48 (21%)
Vegetation	89 (40%)
Abscess	19 (8%)
Combined	29 (13%)
Mitral valve	
Insufficiency (grade I–IV)	184 (82%)
Insufficiency ≥ grade II	104 (46%)
Stenosis	5 (2%)
Combined	3 (1%)
Not affected	32 (14%)
Vegetation	90 (40%)
Abscess	2 (1%)
Combined	7 (3%)
Tricuspid valve	
Insufficiency (grade I–IV)	101 (45%)
Insufficiency ≥ grade 2	45 (20%)
Stenosis	3 (1%)
Combined	-
Not affected	120 (54%)
Vegetation	18 (8%)
Abscess	-
Combined	4 (2%)
Abscess	60 (27%)
Double valve endocarditis	39 (18%)

GFR: glomerular filtration rate; LV: left ventricular, NYHA: New York Heart Association; EuroSCORE II provided in median and interquartile range.

**Table 2:** Identified pathogens.

Pathogens	Occurrence
*Staphylococcus aureus*	72 (32%)
Viridans streptococci (*S. mutans*, *S. mitis*, *S. anginosus*, *S. sanguinis*, *S. salivarius*, *S. gordonii*, *S. constellatus*)	38 (17%)
*Enterococcus faecalis*	31 (14%)
*Staphylococcus epidermidis*	13 (6%)
*Cutibacterium acnes*	11 (5%)
*Streptococcus agalactiae*	9 (4%)
*Granulicatella adiacens*	5 (2%)
*Haemuphilus influencae*	5 (2%)
*Staphylococcus lugdunensis*	5 (2%)
*Streptococcus gallolyticus*	5 (2%)
Others	22 (10%)
*Abiotrophia defectiva*	
*Bartonella quintana*	
*Bartonella henselae*	
*Cardiobacterium hominis*	
*Citrobacterium koseri*	
*Corynebacterium striatum*	
*Enterococcus gallinarum*	
*Serratia marcescens*	
*Staphylococcus capitis*	
*Moraxella osloensis*	
*Clostridium intestinale*	
*Pseudomonas aeruginosa*	
*Streptococcus pneumoniae*	
*Streptococcus dysgalactiae*	
*Staphylococcus saprophyticus*	
*Staphylococcus haemolyticus*	

Most frequently found pathogens and occurrence: *n* (%); others: pathogens which were identified at least two times via PCR and tissue sample.

## Data Availability

Data supporting the reported results can be provided by the first author upon request.

## References

[1] Murdoch D.R., Corey G.R., Hoen B., Miró J.M., Fowler V.G., Bayer A.S., Karchmer A.W., Olaison L., Pappas P.A., Moreillon P. (2009). Clinical Presentation, Etiology, and Outcome of Infective Endocarditis in the 21st Century: The International Collaboration on Endocarditis–Prospective Cohort Study. Arch. Intern. Med..

[2] Delgado V., Marsan N.A., de Waha S., Bonaros N., Brida M., Burri H., Caselli S., Doenst T., Ederhy S., Erba P.A. (2023). ESC Scientific Document Group; 2023 ESC Guidelines for the management of endocarditis: Developed by the task force on the management of endocarditis of the European Society of Cardiology (ESC) Endorsed by the European Association for Cardio-Thoracic Surgery (EACTS) and the European Association of Nuclear Medicine (EANM). Eur. Heart J..

[3] Cahill T.J., Prendergast B.D. (2016). Infective endocarditis. Lancet.

[4] Prendergast B.D., Tornos P. (2010). Surgery for infective endocarditis: Who and when?. Circulation.

[5] Bohbot Y., Peugnet F., Lieu A., Carbone A., Mouhat B., Philip M., Gouriet F., Arregle F., Chevalier F., Diouf M. (2021). Characteristics and Prognosis of Patients with Left-Sided Native Bivalvular Infective Endocarditis. Can. J. Cardiol..

[6] Thuny F., Grisoli D., Cautela J., Riberi A., Raoult D., Habib G. (2014). Infective endocarditis: Prevention, diagnosis, and management. Can. J. Cardiol..

[7] Godfrey R., Curtis S., Schilling W.H., James P.R. (2020). Blood culture negative endocarditis in the modern era of 16S rRNA sequencing. Clin. Med..

[8] Baddour L.M., Wilson W.R., Bayer A.S., Fowler V.G., Tleyjeh I.M., Rybak M.J., Barsic B., Lockhart P.B., Gewitz M.H., Levison M.E. (2015). Infective Endocarditis in Adults: Diagnosis, Antimicrobial Therapy, and Management of Complications. Circulation.

[9] Goldenberger D., Künzli A., Vogt P., Zbinden R., Altwegg M. (1997). Molecular diagnosis of bacterial endocarditis by broad-range PCR amplification and direct sequencing. J. Clin. Microbiol..

[10] Breitkopf C., Hammel D., Scheld H.H., Peters G., Becker K. (2005). Impact of a molecular approach to improve the microbiological diagnosis of infective heart valve endocarditis. Circulation.

[11] Fournier P.E., Thuny F., Richet H., Lepidi H., Casalta J.P., Arzouni J.P., Maurin M., Célard M., Mainardi J.L., Caus T. (2010). Comprehensive diagnostic strategy for blood culture-negative endocarditis: A prospective study of 819 new cases. Clin. Infect. Dis. Off. Publ. Infect. Dis. Soc. Am..

[12] Vollmer T., Piper C., Horstkotte D., Körfer R., Kleesiek K., Dreier J. (2010). 23S rDNA real-time polymerase chain reaction of heart valves: A decisive tool in the diagnosis of infective endocarditis. Eur. Heart J..

[13] Harris K.A., Yam T., Jalili S., Williams O.M., Alshafi K., Gouliouris T., Munthali P., NiRiain U., Hartley J.C. (2014). Service evaluation to establish the sensitivity, specificity and additional value of broad-range 16S rDNA PCR for the diagnosis of infective endocarditis from resected endocardial material in patients from eight UK and Ireland hospitals. Eur. J. Clin. Microbiol. Infect. Dis. Off. Publ. Eur. Soc. Clin. Microbiol..

[14] Miller R.J., Chow B., Pillai D., Church D. (2016). Development and evaluation of a novel fast broad-range 16S ribosomal DNA PCR and sequencing assay for diagnosis of bacterial infective endocarditis: Multi-year experience in a large Canadian healthcare zone and a literature review. BMC Infect. Dis..

[15] Armstrong C., Kuhn T.C., Dufner M., Ehlermann P., Zimmermann S., Lichtenstern C., Soethoff J., Katus H.A., Leuschner F., Heininger A. (2020). The diagnostic benefit of 16S rDNA PCR examination of infective endocarditis heart valves: A cohort study of 146 surgical cases confirmed by histopathology. Clin. Res. Cardiol. Off. J. Ger. Card. Soc..

[16] Habib G., Lancellotti P., Antunes M.J., Bongiorni M.G., Casalta J.P., Del Zotti F., Dulgheru R., El Khoury G., Erba P.A., Iunget B. (2015). 2015 ESC Guidelines for the management of infective endocarditis: The Task Force for the Management of Infective Endocarditis of the European Society of Cardiology (ESC). Endorsed by: European Association for Cardio-Thoracic Surgery (EACTS), the European Association of Nuclear Medicine (EANM). Eur. Heart J..

[17] Geissdorfer W., Moos V., Moter A., Loddenkemper C., Jansen A., Tandler R., Morguet A.J., Fenollar F., Raoult D., Bogdan C. (2012). High frequency of *Tropheryma whipplei* in culture-negative endocarditis. J. Clin. Microbiol..

[18] Gould F.K., Denning D.W., Elliott T.S., Foweraker J., Perry J.D., Prendergast B.D., Sandoe J.A., Spry M.J., Watkin R.W. (2012). Working Party of the British Society for Antimicrobial C. Guidelines for the diagnosis and antibiotic treatment of endocarditis in adults: A report of the Working Party of the British Society for Antimicrobial Chemotherapy. J. Antimicrob. Chemother..

[19] Li J.S., Sexton D.J., Mick N., Nettles R., Fowler V.G., Ryan T., Bashore T., Corey G.R. (2000). Proposed Modifications to the Duke Criteria for the Diagnosis of Infective Endocarditis. Clin. Infect. Dis..

[20] Lohmann C.P., Linde H.J., Reischl U. (2000). Improved detection of microorganisms by polymerase chain reaction in delayed endophthalmitis after cataract surgery. Ophthalmology.

[21] Benito N., Miro J.M., de Lazzari E., Cabell C.H., del Rio A., Altclas J., Commerford P., Delahaye F., Dragulescu S., Giamarellou H. (2009). Health care-associated nativevalve endocarditis: Importance of non-nosocomial acquisition. Ann. Intern. Med..

[22] Habib G. (2003). Embolic risk in subacute bacterial endocarditis: Role of transesophageal echocardiography. Curr. Cardiol. Rep..

[23] Thuny F., di Salvo G., Belliard O., Avierinos J.F., Pergola V., Rosenberg V., Casalta J.P., Gouvernet J., Derumeaux G., Iarussi D. (2005). Risk of embolism and death in infective endocarditis: Prognostic value of echocardiography: A prospective multicenter study. Circulation.

[24] Brouqui P., Raoult D. (2001). Endocarditis due to rare and fastidious bacteria. Clin. Microbiol. Rev..

[25] Moreillon P., Que Y.A. (2004). Infective endocarditis. Lancet.

[26] Raoult D., Birg M.L., la Scola B., Fournier P.E., Enea M., Lepidi H., Roux V., Piette J.C., Vandenesch F., Vital-Durand D. (2000). Cultivation of the bacillus of Whipple’s disease. N. Engl. J. Med..

[27] Bosshard P.P. (2003). Etiologic Diagnosis of Infective Endocarditis by Broad-Range Polymerase Chain Reaction: A 3-Year Experience. Clin. Infect.Dis..

[28] Madico G.E., Rice P.A. (2008). 16S-Ribosomal DNA to Diagnose Culture-Negative Endocarditis. Curr. Infect. Dis. Rep..

[29] Tak T., Shukla S.K. (2004). Molecular Diagnosis of Infective Endocarditis: A Helpful Addition to the Duke Criteria. Clin. Med. Res..

[30] Rothman R.E., Majmudar M.D., Kelen G.D., Madico G., Gaydos C.A., Walker T., Quinn T.C. (2002). Detection of bacteremia in emergency department patients at risk for infective endocarditis using universal 16S rRNA primers in a decontaminated polymerase chain reaction assay. J. Infect. Dis..

[31] Arvieux C., Common H. (2019). New diagnostic tools for prosthetic joint infection. Orthop. Traumatol. Surg. Res..

